# Examples of Inverse Comorbidity between Cancer and Neurodegenerative Diseases: A Possible Role for Noncoding RNA

**DOI:** 10.3390/cells11121930

**Published:** 2022-06-15

**Authors:** Michele Salemi, Maria Paola Mogavero, Giuseppe Lanza, Laura M. Mongioì, Aldo E. Calogero, Raffaele Ferri

**Affiliations:** 1 Oasi Research Institute, IRCCS (Istituti di Ricovero e Cura a Carattere Scientifico), Italian Ministry of Health, 94018 Troina, Italy; giuseppe.lanza1@unict.it (G.L.); rferri@oasi.en.it (R.F.); 2 Istituti Clinici Scientifici Maugeri, IRCCS (Istituti di Ricovero e Cura a Carattere Scientifico), Scientific Institute of Pavia, 27100 Pavia, Italy; paola_mogavero@libero.it; 3 Department of Surgery and Medical-Surgical Specialties, University of Catania, 95123 Catania, Italy; 4 Department of Clinical and Experimental Medicine, University of Catania, 95123 Catania, Italy; lauramongioi@hotmail.it (L.M.M.); acaloger@unict.it (A.E.C.)

**Keywords:** noncoding RNAs, inverse comorbidity, cancer, neurodegenerative diseases

## Abstract

Cancer is one of the most common causes of death; in parallel, the incidence and prevalence of central nervous system diseases are equally high. Among neurodegenerative diseases, Alzheimer’s dementia is the most common, while Parkinson’s disease (PD) is the second most frequent neurodegenerative disease. There is a significant amount of evidence on the complex biological connection between cancer and neurodegeneration. Noncoding RNAs (ncRNAs) are defined as transcribed nucleotides that perform a variety of regulatory functions. The mechanisms by which ncRNAs exert their functions are numerous and involve every aspect of cellular life. The same ncRNA can act in multiple ways, leading to different outcomes; in fact, a single ncRNA can participate in the pathogenesis of more than one disease—even if these seem very different, as cancer and neurodegenerative disorders are. The ncRNA activates specific pathways leading to one or the other clinical phenotype, sometimes with obvious mechanisms of inverse comorbidity. We aimed to collect from the existing literature examples of inverse comorbidity in which ncRNAs seem to play a key role. We also investigated the example of mir-519a-3p, and one of its target genes Poly (ADP-ribose) polymerase 1, for the inverse comorbidity mechanism between some cancers and PD. We believe it is very important to study the inverse comorbidity relationship between cancer and neurodegenerative diseases because it will help us to better assess these two major areas of human disease.

## 1. Introduction

### 1.1. Epidemiological and Social-Healthcare Background

The World Health Organization indicates cancer as one of the most common causes of death, which accounted for almost 10 million deaths worldwide in 2020 [1]. The incidence and prevalence of central nervous system (CNS) diseases are high, as well. Among neurodegenerative diseases, Alzheimer’s dementia (AD), characterized by brain amyloid plaques and neurofibrillary tangles of phosphorylated tau (P-tau) protein deposits, is the most common, affecting 24 million people worldwide. The second most frequent neurodegenerative disease is Parkinson’s disease (PD), characterized by Lewy bodies and neurites of alpha-synuclein deposits. PD has a prevalence of 1% in people older than 60 years and 3% in those aged 80 years or older [2].

Despite being an inflammatory demyelinating disease, multiple sclerosis (MS) can also be viewed as a neurodegenerative condition because of the cascade of events triggered by neuroinflammation, eventually leading to chronic cellular stress and imbalance of ion homeostasis, with axonal and neuronal death [3,4,5]. The total prevalence of MS in Europe is 83 per 100,000, which is lower than the prevalence of AD and PD, but still associated with significant social and healthcare costs [6,7]. Amyotrophic lateral sclerosis, (ALS), another condition worthy of mention, although relatively rare (prevalence of 2–3/100,000), is a progressive degeneration of motor neurons in the brain and spinal cord that has very high clinical and social costs [8].

Multiple health-related conditions are present in almost one-quarter of all patients and in more than half of those with a chronic disorder [9]. The comorbidity of cancer and neurological disorders has been established by a series of observational studies [10,11,12]. For instance, Down’s syndrome (DS) is among the CNS disorders most heavily associated with increased co-occurrence of cancer, such as acute leukemia, testicular tumor and some gastrointestinal cancers [13]. At the same time, however, emerging evidence points to a lower-than-expected probability of some types of cancer in certain CNS disorders [14], in particular, for neurodegenerative diseases such as PD and AD [15], an association termed “inverse cancer comorbidity” [16]. More precisely, inverse comorbidity is a lower-than-expected probability of a disease occurring in people who have other diseases; this phenomenon is also influenced by dietary, environmental and pharmacological factors, among others [17,18].

Inverse comorbidity has been reported in patients with PD, specifically colorectal and prostate cancers [13,19]. Establishing the co-occurrence of cancer in individuals with CNS disorders, and vice versa, is a crucial step toward the development of effective preventive and therapeutic strategies for both disease groups [20,21,22]. Understanding why and how people with certain neurodegenerative disorders are protected against some types of cancer might be the key to developing novel treatments for both conditions.

To date, neurodegenerative diseases and cancers are still considered to be pathogenically and clinically distinct, although they actually have intriguing interrelationships. Epidemiologically, both AD and PD are less frequent in survivors of many cancers, and vice versa, thus suggesting that a propensity toward one group of diseases may decrease the risk of the other [13,14,23,24]. Similarly, many cancer survivors are at higher risk of some non-neurodegenerative disorders, such as stroke, vascular dementia and macular degeneration [25,26]. Nevertheless, the inverse association is not consistent across all cancer types, with an increased risk of malignant melanoma in patients with PD the most remarkable example [27,28].

Cancer treatment may modify this relationship, with some studies suggesting that chemotherapy-treated breast cancer survivors may have less white matter organization and connectivity when compared to healthy controls [29], and others associating chemotherapy with a lower risk of subsequent AD [26]. Thus, epidemiological associations are actually rather complex, and they are challenged by the difficulty of accounting for the ways in which diagnosis, treatment and survival from one disease influence the risk of the other [30]. A previous meta-analysis of cancer incidence in 577,013 participants recruited by 50 observational studies [31] found that the presence of CNS disorders was associated with a reduced co-occurrence of cancer, and a consistently lower co-occurrence of cancer was detected in patients with neurodegenerative disorders. Patients with DS had a higher co-occurrence of cancer, whereas no association was observed between cancer and amyotrophic ALS. Of note, patients with PD and MS showed a greater co-occurrence of certain cancers (e.g., PD with melanoma and MS with brain cancers) and lower co-occurrence of other cancers (e.g., lung, prostate and colorectal cancers in PD; lung and prostate cancers in MS) [31].

Inverse comorbidity has its biological basis in a likely complex mechanism, in which more general and ubiquitous factors and processes, such as reactive oxygen species, microRNA, mitochondrial function, etc., as well as some very specific ones, may play a combined role with different weights, with the result being to favor neurodegeneration or cancer, alternatively or in mutual exclusion [32].

### 1.2. Biological Connections between Cancer and Neurodegeneration

**Figure 1:**
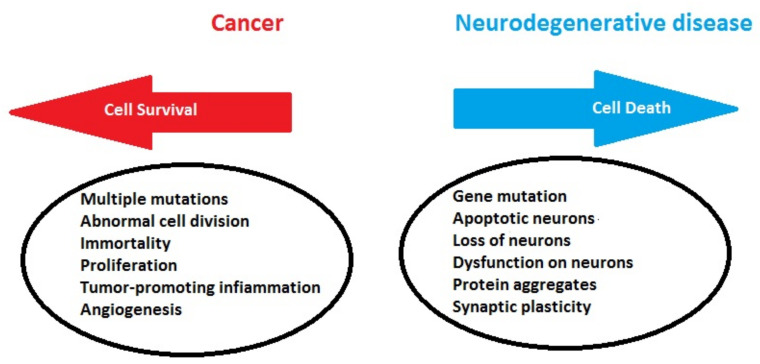
General biological mechanisms leading to neurodegenerative disease or cancer.

An expanding body of literature describes genes, proteins and pathways dysregulated in both cancers and neurodegenerative diseases, often in opposite directions. Among them, the expression of p53, a well-known tumor-suppressor gene, is upregulated in AD, PD and HD [33,34,35,36] but downregulated in a large majority of cancers [37]. *PIN1*, a multifunctional gene hypothesized to act as a “molecular timer”, is upregulated in a number of cancers and downregulated in AD [38,39]. On the other hand, there is a substantial positive pathophysiological overlap, with oxidative stress, DNA damage, inflammation, metabolic deregulation and aberrant cell cycle activation playing central roles in both diseases (Figure 1).

In this context, it is now well-established that aging is a manifestation of the time-dependent accumulation of cellular damage [40], and several key cellular and molecular hallmarks of physiologic aging have been identified, such as genomic instability, telomere attrition, epigenetic alterations, loss of proteostasis, deregulated nutrient sensing, mitochondrial dysfunction, cellular senescence, stem cell exhaustion and altered intercellular communication [41], which are all highly involved in both carcinogenesis and neurodegeneration. One of the reasons underlying this increasing interest in physiologic aging lies in the fact that genetic diseases commonly have an early age of onset and lead to early disability and premature death; as a result, they remain rare in the population [42].

In contrast, chronic age-related diseases, such as cancer and neurodegenerative disorders, usually have complex causes that include small contributions from many genes, as well as aging-related changes and the contribution of different environmental and lifestyle factors [43,44,45,46,47,48]. As an example, the connections between the genetics of PD and cancer can be considered: while the familial PD genes are used by neurons to control protein processing and clearance, most also play some role in development or cell cycle regulation in dividing cells. Among them, PARK2 (Parkin) and PARK5 are critical parts of the ubiquitin-proteasome system (UPS), which is the main pathway by which proteins are degraded in cells. These genes have anti-proliferative properties and are often inactivated in cancers [49,50]. *PARK6* may also have anti-proliferative functions [51,52]. Thus, these genes are both neuroprotective and tumor-suppressive. Meanwhile, PARK7 (DJ–1), although taking part in the UPS, antagonizes the tumor-suppressor gene *PTEN* in dividing cells [53,54,55]. As such, rather than functioning as a tumor suppressor, *PARK7* may be considered an oncogene. In this way, there are “mixed signals”, which may explain, at least partially, why there are both positive and negative associations between PD and cancer.

Less is known about the shared genetic component between AD and cancer, mainly because strongly AD-associated genes are not known oncogenes or tumor suppressors. Using summary statistics from large genome-wide association studies, recently, evidence has been found of a shared genetic component between AD and five cancers (i.e., colon, breast, prostate, ovarian and lung [56]), suggesting that gene-expression regulators play an important role in the genetic overlap. Moreover, some shared variants may modulate the risk of both diseases in the same direction, while others increase the risk of one disease while decreasing the risk of the other [42].

As mentioned above, *PIN1* has been recently proposed as a novel and key regulator at the crossroad between cancer and AD [57]. PIN1 is a peptidyl-prolyl cis-trans isomerase that catalyzes cis-trans isomerization, regulating the conformation of different protein substrates after phosphorylation and thus modulating protein function [58]. In particular, trans-conformations of the amyloid precursor protein (APP) and P-tau are normally functional, while cis-conformations, triggered after phosphorylation, are pathogenic [59]. PIN1 accelerates APP cis-to-trans isomerization, thus favoring the non-amyloidogenic pathway, while, in the absence of PIN1, APP is processed through the amyloidogenic pathway, thus predisposing an individual to AD. Furthermore, when PIN1’s function is inhibited, P-tau is hyperphosphorylated [59], and data from brain specimens have revealed very low PIN1 expression in these patients. Finally, polymorphisms in the *PIN1* promoter have been correlated with increased PIN1 expression and associated with a delay in the sporadic AD age of onset, while reduced *PIN1* expression is associated with a decreased risk of multiple cancers [60].

Translationally, these findings have a number of relevant implications for preventive and therapeutic approaches to both cancer and neurodegeneration. First, interventions that slow the hallmarks of aging will decrease the risk of both groups of diseases. It has been consistently shown that a healthy lifestyle and metabolism can decrease the risk of both cancer and cognitive decline [61,62,63,64,65,66]. In addition, related interventions are known to decrease markers of inflammation, improve mitochondrial health and decrease oxidative stress [67,68]. Medications targeting various hallmarks of aging are also being explored in both fields. For instance, metformin, a biguanide for the treatment of type 2 diabetes, appears to decrease the risk of cancer, dementia and other age-related diseases through multiple mechanisms [69,70], including decreased levels of insulin and IGF–1, inhibition of the mTOR pathway, inhibition of mitochondrial function, a decrease in oxidative damage and activation on the AMP kinase [71,72,73,74].

Senescent cells are an ideal target for therapy because of their presence in many age-related diseases, including both carcinogenesis and neurodegeneration. Drugs that have been tested in cancer for years are now being actively investigated as neuroprotective agents [42]. Therapies targeting proteostasis involving these genes are being explored in animal models of AD due to the altered excretion of toxic metabolites, mainly caused by aberrant mutations in autophagy-associated genes [75]. In another strand of work, senescent cells express unique proteins that cause intriguing age-related deterioration and thus can be used as potential targets in new therapies [76].

Finally, proteasome inhibitors, such as bortezomib, inhibit protein degradation, and their role as anti-cancer agents has been expanding in the past decade. If inhibitors of the proteasome are effective anti-cancer agents, then drugs that enhance proteasome function will likely be neuroprotective [42]. Accordingly, small molecules that increase proteasome 26S activity are currently being developed for this purpose [77]. However, proteasome activation needs to be carefully targeted to neurons as it could theoretically promote cancer in other tissues, whereas proteasome inhibition should theoretically cause PD, although to date there is no evidence of this. Inhibitors targeting heat shock proteins have been in development for cancer for years, although work to modulate the chaperone network of proteins as a neuroprotective strategy has begun only recently [78]. Lastly, modulating a single chaperone protein, such as Hsp90, has been proposed as a potential therapy for both cancer and neurodegeneration [79].

### 1.3. Aim and Hypothesis

Noncoding RNAs (ncRNAs) are defined as transcript nucleotides that carry out a number of functions, including transcriptional regulation, organization of nuclear domains and stabilization of proteins or RNA molecules [80]. The mechanisms through which ncRNAs exert their functions are numerous and involve every aspect of cell life [81]. The same ncRNA can act in multiple ways, leading to different outcomes: a single ncRNA can participate in the pathogenesis of more than one disease, even if these seem very different, as are cancer and neurodegenerative disorders. The ncRNA activates specific pathways that lead to one or the other clinical phenotype [82]. Although their outcomes are very different, the pathways involved in cell proliferation and loss of cell differentiation in cancer and progressive neuronal cell death in neurodegeneration may be similar, though working under a different modulation mechanism [83]. Mutations in a variety of genes involved in the regulation of the cell cycle, DNA repair, protein turnover, oxidative stress and autophagy have been implicated in both clinical phenotypes [84]. Among the mechanisms involved, alterations to RNA metabolism are obtaining significant attention given the critical role of RNA transcription, maturation, transport, stability, degradation and translation in cellular functions [83]. As a matter of fact, some of the most known ncRNAs, such as NEAT1 [85,86], HOTAIR [87,88] and MALAT1, participate in both groups of disorders, where they exert different functions. For instance, MALAT1 is upregulated in many types of cancer, driving tumor progression by regulating tumor cell proliferation, metastasis and migration [89]. It is upregulated in myocardial infarction [90], exerts a protective role in ischemic stroke [91] and also participates in PD, where it increases the stability of α-synuclein [92]. Therefore, accumulating evidence demonstrates that long-noncoding RNAs (lncRNAs) may affect the pathogenesis of several diseases, especially cancers and neuroimmunological and neurodegenerative disorders [93,94,95,96].

In this review, we further examine the biological and genetic overlap between cancer and neurodegeneration, attempting to develop new lines of thought to elucidate the mixed signals that underlie this complex relationship. One of these is the inverse association involving ncRNAs and their target genes in inverse comorbidity mechanisms. While some of the connections between these disorders might help explain the inverse comorbidity pattern seen in epidemiological studies, others suggest that the diseases should co-occur. This mixed picture may help to explain why an inverse association with neurodegeneration is seen in some cancers but not others. Both positive and inverse associations in overlapping biology will provide potential new directions for developing effective prevention and treatment. Therefore, we have set out in this Review some examples of inverse comorbidity in which ncRNAs, and in some cases, their target genes, are involved, with the aim to stress to the scientific community the importance of this aspect, as demonstrated using clear experimental data.

## 2. Classification of Noncoding RNAs

**Figure 2:**
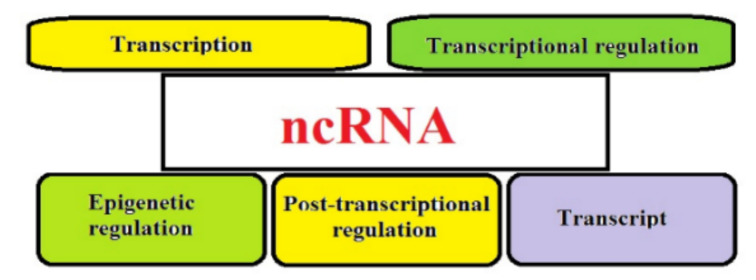
Gene regulatory mechanism of ncRNA.

ncRNAs are RNA molecules that are transcribed but not translated into proteins; they perform multiple biological functions by acting on target nucleotide sequences through sequence-specific interactions and play a regulatory role in many biological processes, protecting genomes from foreign nucleic acids, guiding DNA rearrangement or synthesis in the genome and playing a crucial role in gene expression (RNA processing, transcription and translation) (Figure 2) [97].

The biogenesis of different types of ncRNAs has been described by various authors [98,99,100,101,102]. ncRNAs are classified according to function or size; with the former approach, ncRNAs are classified as translation-related ncRNAs or housekeeping ncRNAs. The ncRNAs that play a regulatory role include ncRNA, small endogenous interfering RNA (siRNA), PIWI-interacting RNAs (piRNAs) and micro RNA (miRNA), while those that are constitutively expressed and play a crucial role in normal cell function and viability are small nucleolar RNA (snoRNA), siRNA, rRNA and tRNA [98,99].

**Figure 3:**
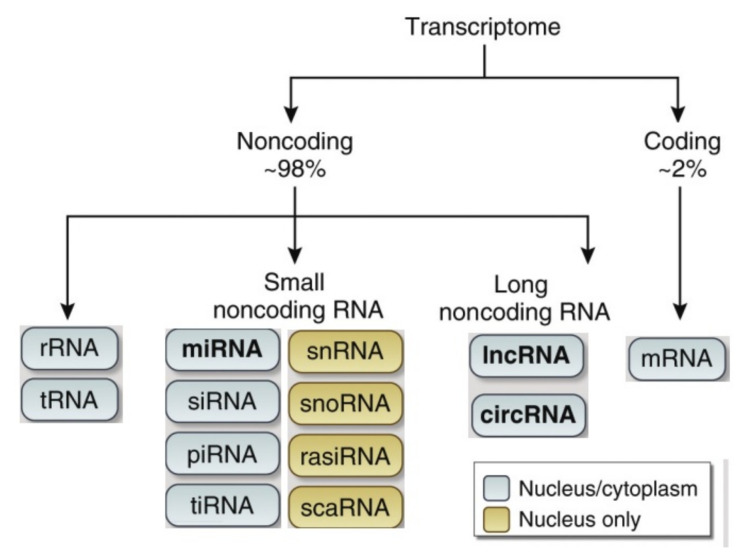
RNAs’ nomenclature and function. RNAs are classified based on their coding. Only two percent of RNAs code for proteins; most RNAs exert regulatory functions. lncRNA, long noncoding RNA; circular RNA; miRNA; microRNA; piRNA; piwi-interacting RNA; rasiRNA; repeat-associated small interfering RNA; scaRNA, small Cajal body-specific RNA; siRNA, small interfering RNA; snRNA, small nuclear RNA; snoRNA, small nucleolar RNA; tiRNA, transfer RNA; rRNA, stress-induced small RNA; tRNA, transfer RNA.

According to size, ncRNAs are classified primarily for their length. ncRNAs that are classified as small or short are <200 nucleotides in their mature forms. This group includes small nuclear RNA (snRNA), miRNA, piRNA, siRNA and snoRNA. siRNAs are particularly interesting because they consist of double-stranded RNA and act in gene silencing, although they undergo various modifications before acting [103]. Chemical modifications of siRNAs have been shown to improve their intrinsic properties [104]. In contrast, ncRNAs >200 nucleotides in length in their mature forms are classified as lncRNA (Figure 3) [98,99].

Long noncoding RNAs (lncRNAs) are based on a length threshold of 200 nucleotides [100]. While lncRNAs cannot encode proteins like mRNAs, they may participate in the progression of various diseases through other mechanisms by acting on the regulation of mRNAs [105]. Furthermore, lncRNAs have been shown to influence epigenetic regulation [106], DNA damage [107], the cell cycle [108] and chromosomal instability, suggesting their potential role in oncological and neurodegenerative mechanisms. One particular class of ncRNAs is the circular RNAs (circRNAs), which function as sponges to control miRNA levels [109]. CircRNAs are formed through a back-splicing process from mRNAs that encode proteins through canonical splicing [110,111]. Since these are continuous closed rings that lack defined 5′ caps and 3′ poly-A tails, they are resistant to RNase R. Therefore, the circRNAs are incredibly stable, with a half-life of more than 48 h, which is long when compared to the corresponding linear RNAs [111,112].

## 3. Mechanisms of Inverse Comorbidity between Cancer and Parkinson’s Disease: Mir-519a-3p and Its Interactions with the PARP1 Gene

### 3.1. Mir-519a-3p and Cancer

miRNAs are 19- to 25-nt transcripts that originate from 70- to 100-nt precursors, and are encoded in the genomes of plants, invertebrates and vertebrates [113]. The sequence of many miRNAs is conserved among organisms that are distant from an evolutionary point of view, which suggests that these molecules play an important role in essential processes [114]. The biological functions of miRNAs are only partially understood; certainly, miRNAs play crucial roles in cell proliferation, cell death, developmental stages of organisms, stress resistance and various aspects of metabolism [115]. Moreover, there are several indications that miRNAs might be involved in human tumorigenesis [116,117,118,119]. In this review, we want to emphasize the role of miR-519a-3p in both oncological and neurodegenerative processes. Glioblastoma (GB) is the most aggressive brain tumor [120]; 3 out of every 100,000 people develop GB each year, and the five-year survival rate is less than 5% [121]. Chemotherapy, especially with temozolomide (TMZ), seems to be an effective treatment method for GB [122,123], but unfortunately for the prognosis of the disease, there are mechanisms of TMZ resistance that seriously limit its use for the treatment of GB [124,125]. TMZ-resistant GB cells have a high capacity for cell invasiveness and metastasis creation [124,125]. It has been repeatedly reported that microRNAs and lncRNAs are involved in the regulation of chemoresistance in many cancers [126,127,128,129]; for example, miR-519a-3p influences the sensitivity of GB cells to TMZ chemotherapy [130]. Specifically, miR-519a-3p performs its action in conjunction with HOTAIR lncRNA [131], which is significantly upregulated in TMZ-resistant GB cells, while its downregulation inhibits proliferation, migration, invasion and epithelial/mesenchymal transition to TMZ-resistant GB cells. A further analysis demonstrated that HOTAIR lncRNA induces TMZ resistance through miR-519a-3p [131]; however, the specific mechanism with which miR-519a-3p regulates TMZ resistance in GB is not well understood. Other studies have shown that miR-519a-3p is linked to tamoxifen resistance in breast cancer [132] and cisplatin resistance in non-small cell lung cancer [133].

In a study by Flor et al. [134], it was shown that miR-519a-3p is more greatly expressed in normal testicular tissue than in other normal adult tissues, but more importantly, miR-519a-3p is also highly expressed in testicular germ cell tumors (TGCTs). The authors found miR-519a-3p significantly overexpressed in non-seminomas, which are more aggressive than seminomas, and which have an earlier age of onset [135]; moreover, miR-519a-3p expression was shown to be higher in more advanced stages of tumors, suggesting that miRNAs might be involved in the greater aggressiveness of TGCTs and metastasis formation. Novotny et al. [136], using microarrays to examine the expression of miRNAs in seminomas, observed the upregulation of miR-519a-3p in embryonal carcinomas (ECs), seminomas and unclassified intratubular germ cell neoplasm (ITGCNU) compared to normal testes.

The most aggressive forms of breast cancer are associated with poorer patient outcomes [137,138] and are characterized by the development of cancer cell clones that can resist targeted therapies or escape the control of the immune system [139,140]. The complex molecular mechanisms that lead to immune escape and resistance to therapies are not fully understood; in breast cancer, it has been shown that high levels of miR-519a-3p are associated with poorer survival [132,141]. Subsequently, it was shown that miR-519a-3p inhibits the apoptotic mechanism controlled by Fas ligand, (TNF)-related apoptosis-inducing ligand (TRAIL) and granzyme B/perforin interfering in pro-apoptotic signals in breast cancer cells [142]. Overexpression of miR-519a-3p inhibits the expression of its direct target genes for TRAIL-R2 (TNFRSF10B) and caspase-8 and its indirect target gene for caspase-7; this leads to reduced apoptosis of cancer cells in response to the same apoptotic stimuli [142]. Another important finding is that miR-519a-3p inhibits tumor cell killing by natural killer (NK) cells acting on the ligands UL-16 binding protein (ULBP2), NK cell group 2 receptor D (NKG2D) and major histocompatibility complex class I-related chain A (MICA) that are located on the surface of tumor cells; these ligands are crucial for the detection of tumor cells by NK cells. Furthermore, the miR-519a-3p is overexpressed in the most aggressive TP53 mutant breast cancer and thus correlates with poor survival [142]. Hepatocellular carcinoma (HCC) is considered the third-leading cause of death for cancer [143]; from a molecular perspective, HCC is a highly heterogeneous tumor. Several studies have shown abnormal miRNA expression in HCC, with upregulation of miR-221, miR-21 and miR-151, and downregulation of miRNA Let-7, miR-29, miR-122 and miR-26a [144,145,146,147]. In the study by Toffanin et al. [148], it was shown that miR-519a-3p also appears to be overexpressed in HCC.

### 3.2. miR-519-3p in Parkinson’s Disease

PD is a progressive neurodegenerative disorder, characterized by a loss of dopaminergic neurons in the substantia nigra pars compacta (SNpc) with a decrease in dopamine, which leads to the classic motor symptoms of rigidity, bradykinesia and tremor [149]. A large proportion of PD cases are believed to be sporadic (sPD) and are hypothesized to be the result of an interaction between genetic and environmental factors, in addition to aging, which is considered the most important risk factor [150]. A small percentage of PD patients present monogenic hereditary forms caused by pathogenic gene mutations [151]; of these, the majority are missense mutations located in the leucine-rich repeat kinase 2 (LRRK2) gene. Importantly, LRRK2 mutations have been identified not only in LRRK2-associated PD but also in many cases of sPD, supporting a reduced penetrance driven by other factors [152].

miRNA alterations have been shown to contribute to the pathophysiology of neurodegenerative disorders, including PD [153,154]. Tolosa et al. [155] identified 10 differentially expressed miRNAs; five were downregulated (miR-141e3p, miR-299e5p, miR-199a-5p, miR-518e-3p and miR-519a-3p) and five were upregulated in PD (miR-9-5p, miR-135b-5p, miR-135a-5p, miR-449a and miR-449b-5p). In this study, performed on miRNAs extracted from skin biopsies, the authors showed that miR-519a-3p is particularly downregulated in subjects with PD, with a –17-fold change compared to the control group. The authors explored biological enrichment of target genes of differentially expressed miRNAs using the DIANA-miR Path v3.0 software; miR-519a-3p was shown to influence target genes that play a role in specific neural functions, such as neural projection, neural differentiation and axogenesis [155]. In the same study, the downregulation of miR-199a-5p was associated with upregulation of genes NELL2, ZIC1, OTX1 and DCC [155]. It has also previously been reported that ZIC1, NELL2, OTX1 and DCC are involved in neural function and show upregulation in PD [156].

### 3.3. Interactions between miR-519-3p and PARP1 Gene

Poly (ADP-ribose) polymerase 1 (*PARP1*) is a 116 kDa nuclear enzyme composed of an N-terminal DNA-binding domain, an auto-modification domain and a C-terminal catalytic domain [157,158]. The catalytic domain can catalyze the formation of poly(ADP-ribose) (PAR) polymers, alter the physicochemical properties of their substrates and regulate several pathways, including protein stability, DNA damage response and cell death [159]. *PARP1* plays a role in the regulation of gene expression by acting as a negative controller and a positive factor in transcription [160,161]. *PARP1* is a crucial enzyme in DNA repair and modulating the cellular response to stress. It plays a dual role: it is a critical enzyme in DNA repair [162] and can cause cell death by parthanatos (PARP1-dependent cell death, a form of programmed cell death) [163,164].

The role of PARP1 in triggering DNA repair processes in tumor development is well known [165], as well as its upregulation in various cancers [166]; indeed, two of our studies demonstrated that the *PARP1* protein was overexpressed in both prostate cancer [167] and glioblastoma multiforme nuclei [168]. *PARP1* has implications not only in cancer but also in neurodegenerative diseases [169]; activation of PARP1 has been linked to PD pathogenesis in a mouse model, and changes in PAR polymers present in cerebrospinal fluid and brain homogenates of PD patients have been reported [170]. In another of our immunofluorescence and immunohistochemistry studies on postmortem human brains of PD patients and control subjects, PARP1 protein expression was assessed. In control subjects, PARP1 revealed intense nuclear and cytoplasmic staining in pigmented neurons of the substantia nigra. In contrast, in patients with PD, *PARP1* staining was decreased in both the nucleus and cytoplasm of pigmented neurons of the substantia nigra [171].

*PARP1* is upregulated in ovarian cancer (OC) [172,173]. Chang et al. [174] investigated the interactions between miR-519-3p and *PARP1,* in addition to studying their role in OC, and confirmed the role of *PARP1* in OC, along with the expression of *PARP1* in OC tissues and in the adjacent normal tissue, by real-time polymerase chain reaction. These experiments showed an overexpression of *PARP1* compared to normal tissues. To confirm the results, a luciferase reporter double assay was conducted using WT-PARP1-3′-UTR containing miR-519a-3p and PARP1-3′-UTR-mut binding sites. These experiments demonstrated that miR-519a-3p binds to the PARP1- 3′-UTR site [174].

### 3.4. Considerations on miR-519a-3p and its Interactions with the PARP1 Gene

**Figure 4:**
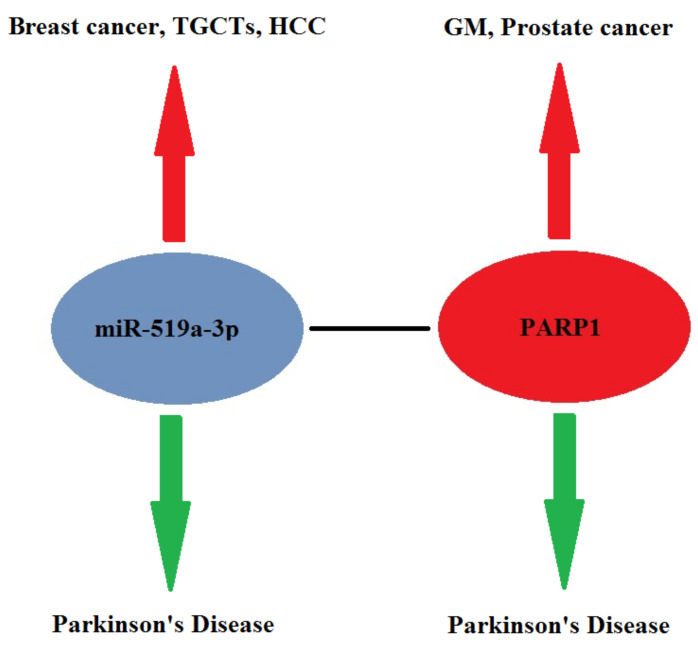
Examples of inverse comorbidity between tumors and Parkinson’s disease in which the miR-519a-3p and PARP1 gene play an important role. TGCTs, testicular germ cell tumors; HCC, hepatocellular carcinoma; GM, glioblastoma multiforme. Red arrow, upregulated; green arrow, downregulated.

As demonstrated in this section, miR-519a-3p seems to be upregulated in some cancers, such as breast cancer, TGCTs and HCC; on the other hand, it is clear that miR-519a-3p is downregulated in PD. These data highlight that miR-519a-3p can be considered an example of an ncRNA that can play a role in the inverse comorbidity mechanisms between tumors and PD. Studies carried out on *PARP1* gene expression show that the PARP1 protein tends to be overexpressed in many tumors; on the contrary, the same PARP1 protein is underexpressed in neurodegenerative diseases, such as PD. This suggests a possible inverse comorbidity between tumors and neurodegenerative diseases in which the PARP1 gene is among the protagonists (Figure 4).

We find it very interesting, although the mechanisms are not well understood, that the PARP1 gene, which is a target of miR-519a-3p, along with miR-519a-3p itself, can express in the same direction in some cancers and in opposite ways in PD (Figure 4). We are of the opinion that these mechanisms should be better and deeply investigated in future studies.

## 4. LncRNA and circRNA in Neurodegenerative Diseases and Cancer: Examples of Inverse Comorbidity

LINC00487 is a lncRNA that has been shown to be downregulated in a transcriptome study of PD subjects [94]. On the other hand, LINC00487 was found to be upregulated in squamous cell carcinoma of the lungs [175]. LINC00487 has been shown to be particularly downregulated in PD, and we believe this is another example of inverse comorbidity between cancer and PD subjects [176,177].

Some authors have studied the expression of HOTTIP lncRNA in an in vitro model of ALS, represented by the SH-SY5Y cell line, and found downregulation of HOTTIP, as opposed to its upregulation in glioma and nasopharyngeal carcinoma, as well as in oral tongue squamous cell carcinoma and gastric cancer [178,179]. It can be speculated that HOTTIP lncRNA might have a role in the molecular mechanisms that induce oncogenesis versus neurodegeneration. Based on these observations, the authors hypothesized a “paradigm” in which deregulation of HOTTIP is the opposite in cancer and neurodegeneration.

The circRNA ciRS-7 was first identified by Hansen in 2011 [180]. Subsequently, it was discovered that ciRS-7 plays a role as an oncogene and plays a key role in various types of cancer. The upregulation of ciRS-7 can promote tumor cell invasion and metastasis in several cancer types, including lung cancer [181], hepatocellular carcinoma [182], cervical cancer [183], pancreatic ductal adenocarcinoma [184] and laryngeal squamous cell carcinoma [185]. ciRS-7 is an abundant circRNA in the human brain but was shown to be downregulated in the brains of AD patients [186].

## 5. ncRNA in Prostate Cancer and Neurodegenerative Diseases

Prostate cancer is among the most frequently diagnosed cancers; it is estimated that 233,000 men are diagnosed with prostate cancer annually in the USA. Prostate cancer is also the second-leading cause of cancer death, with 29,000 deaths annually [187,188]. Interestingly, African American men are 1.6 times more likely to develop prostate cancer than European Americans, and the risk of death is 2.4 times higher [187]. Although many social and economic factors have been called on to explain these data, even after adjusting for these factors, the differences remain [189]; therefore, there are unknown mechanisms underlying the development and progression of prostate cancer.

Among ncRNAs, several miRNAs are involved in the development of prostate cancer; for example, as shown above, miR-34a-5p is a tumor-suppressive miRNA implicated in pancreatic cancer, hepatocellular carcinoma and prostate cancer, as well [190,191]. It negatively regulates the transcription of proteins, such as hypoxia-inducible factors 1 A (HIF1A), insulin-like growth factor-binding protein 2 (IGFBP2) and phosphatidylinositol-4,5-bisphosphate 3-kinase catalytic subunit beta (PIK3CB) [191,192]. These proteins are components of the mTOR (mammalian target of rapamycin) and VEGF (vascular-endothelial growth factor) signaling pathways that are involved in prostate cancer development. miR-34a-5p downregulation in prostate cancer is responsible for the overexpression of HIF1A, IGFBP2 and PIK3CB, associated with tumor development and progression [191]. The receptor tyrosine kinase Axl also is downregulated by miR-34a-5p, and its overexpression is associated with cancer genesis and progression and inhibition of apoptosis [190]. Thus, miR-34a-5p/HIF1A, miR-34a-5p/IGFBP2 and miR-34a-5p/PIK3CB, as well as Axl, could represent possible biomarkers and therapeutic targets for prostate cancer; conversely, miR-34a-5p upregulation is associated with the development of AD [193,194].

miR-34a-5p is indeed strongly related to cognitive dysfunction, and it is highly expressed in the brain tissue of patients with AD who underwent autopsy [194]. The exact role of this miRNA in the pathogenesis of AD is not well-understood; it seems to promote the amyloid processing of amyloid precursor proteins (APPs) [194]. Confirming this, Jian and colleagues [195] showed that miR-34a expression increases with aging in the animal model of AD and is upregulated during AD progression; furthermore, Aβ increased with the increase in miR-34a levels. On the contrary, amyloid plaques’ formation and astrogliosis (signs of AD progression) decreased, while cognitive function improved in miR-34a knockout mice, thanks to inhibiting the amyloidogenic processing of APP [195]. The same group of researchers found that synaptic plasticity was promoted by upregulation of the α-amino-3-hydroxy-5-methyl-4-isoxazolepropionic acid (AMPA) and N-methyl-d-aspartate (NMDA) receptors [193], allowing them to conclude that miR-34a could contribute to AD development as it is involved in synaptic deficiency by inhibiting AMPA and NMDA receptor expression.

Another miRNA for which an inverse comorbidity relationship between prostate cancer and AD has been found is miR-125b [196,197], which is differentially expressed in a tissue-specific manner and has both anti-proliferative and pro-apoptotic activity or tumor-promoter function [197]. In prostate cancer, miR-125b expression is downregulated, and this results in promoting cancer progression [197]; a very recent study showed that the levels of miR-125b expression in urine extracellular vesicles, clarified urine and blood plasma are a potential diagnostic marker and therapeutic agent for prostate cancer management [198]. Upregulation of miR-125b was instead found post-mortem in the cerebral grey matter of patients with AD [196,197,198,199]. This miRNA is associated with neuroinflammation and oxidative stress, and the activation of NF-kB, a pro-inflammatory transcription factor, is responsible for its overexpression in AD [199,200]. Moreover, synapsin II mRNA is among the targets of miR-125b, which, consequently, means it could be responsible for the synapsin protein deficit present in the brains of patients with AD [200].

Conflicting results have been reported on miR-107 and prostate cancer. In AD, this miRNA is downregulated and related to the plaque burden and APP cleavage. Mir-107 also seems to be downregulated in prostate cancer, and this is associated with increased expression of granulin, a mitogen and growth factor. However, some studies reported high levels of miR-107 in the urine of men with prostate cancer, suggesting its upregulation [200]; at this point, further studies are needed. In 2016, Liu and colleagues [201] found upregulation of lncRNA PVT1 in prostate cancer; PVT1 promotes the development and progression of cancer by inducing methylation of the miR-146a promoter, thus inhibiting its expression. PVT1 could be used as a prognostic marker of prostate cancer as higher levels are associated with an advanced tumor stage and poor overall and disease-free rates of survival [202]. lncRNA PVT1 has been reported to be involved in the pathogenesis of PD [203]. Reduced expression of PVT1 has recently been found in patients with PD, and this downregulation was responsible for modulation of the epidermal growth factor receptor pathway, resulting in the induction of apoptosis and inhibition of the cell cycle [203].

## 6. ncRNAs and Inverse Comorbidity between Cancer, Neurodegenerative Diseases and Circadian Mechanisms

### 6.1. Inverse Correlation between Cancer and Neurodegenerative Diseases

In recent years, reports in the literature have shown an increasing correlation between cancer and neurodegeneration [42], often through a mechanism of inverse comorbidity, which could be based on mitochondrial dysfunction, alteration of proteostasis, production of oxygen free radicals, and the functioning of some miRNAs [32]. A definite correlation has also emerged between sleep disorders and cancer, both in adult and pediatric patients [204,205], especially circadian rhythm disorders. In fact, the World Health Organization–International Agency for Research on Cancer, when evaluating the experimental and epidemiological evidence of the association between shift work and various cancers, classified “shift work with circadian interruption” as “probably carcinogenic to humans” [206,207].

In humans, the circadian rhythm is controlled by the main regulatory clock, located in the suprachiasmatic nucleus of the hypothalamus, which, in turn, synchronizes the peripheral clocks. Growing evidence suggests that genes involved in the regulation of circadian rhythms also play very important roles in various pathological conditions, including neurodegenerative processes and carcinogenesis [208,209]. Considering the prognostic and therapeutic implications of miRNAs in some neurodegenerative diseases [210,211,212] and cancer [213,214], and the mechanisms of inverse comorbidity found between these two different general pathological processes, it is, therefore, important to focus our attention on the possible role of miRNAs in the regulation of circadian processes in these mechanisms of inverse comorbidity.

### 6.2. Therapeutic Implications of miRNAs in the Processes of Carcinogenesis, Neurodegenerative and Circadian Mechanisms through Inverse Comorbidity

In recent years the usefulness of miRNAs has come to be considered greater than that of other therapies as they are easily manipulated molecules that can perform their action (also reversibly) and that work in a targeted manner against target cells. Therefore, they support a greater efficacy of immunotherapy, with fewer side effects. However, although their use in the therapeutic field is promising and in continuous expansion, there are many limitations, such as in vivo and in vitro stability, the cost of large-scale production and the efficiency of in situ transduction [215]. To overcome this limitation, researchers recently clarified how carcinogenesis occurs in the context of biomolecular condensates: condensates are membrane-free bodies, often formed by liquid-liquid phase separation, which compartmentalize proteins and RNA molecules with related functions, and which can influence the pharmacodynamics of chemotherapeutic agents, give they can overcome the aforementioned limitations and drug-resistance mechanisms [216]. A recent study has shown that the formation of condensates, using the regulation of circadian rhythms, could impact biological processes in plants and other organisms [217], with possible therapeutic implications.

In light of the evidence from the literature, it seems useful to evaluate which miRNAs are involved in both biological processes, with a mechanism of inverse comorbidity, restricting the evaluation to the molecules that interact with the genes and proteins involved in circadian rhythms, to better understand future prospective therapeutics in the field of precision medicine.

A systematic review collected the studies conducted over the past 10 years on the expression of miRNAs and proteins in AD, highlighting 249 inverse relationships between miRNA and proteins in 28 common pathways (which represent new potential therapeutic targets). This meta-analysis revealed: a constant downregulation of miR-132-3p and miR-16 in the advanced stage of AD; no inverse relationships between miR-132-3p and circadian proteins; an inverse relationship between the downregulation of miR-16 and the upregulation of the CLOCK protein of the circadian rhythm; an upregulation of heat shock protein A-4L (HSPA4L); an inverse relationship between casein kinase 1 (CSNK1) overexpression and miRNAs involved in circadian rhythm pathways [218]. The authors of this study, therefore, envisage miRNA-based therapies to regulate circadian rhythms and hippocampal signaling pathways in AD, whereby the daily mid-afternoon administration of a miR-16 mimic with a short half-life degrades CLOCK mRNA transcripts (which are increased in AD), reducing their cellular concentration. In turn, the suppression of CLOCK/BMAL1 reduces the rate of Aβ production and stabilizes the regulation of intracellular calcium levels. Furthermore, the late afternoon administration of miRNA miR-329-3p or miR-495-3p could suppress CSNK1e production and indirectly reduce CLOCK and BMAL1 levels [218].

Although, here, we do not propose new applications for the involvement of miR-132-3p in AD pathology or its potential as a therapeutic target, it should be highlighted that MiR-132-3p is a neuron-specific miRNA associated with synaptic morphogenesis, neuronal growth and hippocampal formation, and its downregulation has been associated with overexpression of the pro-apoptotic genes FOXO3a, EP300 and PTEN [218]. Recent studies have also shown that PI3K-PTEN dysregulation leads to mTOR-mediated upregulation of BMAL1 in normal and malignant epithelial cells [219] and that short- and long-term PTEN depletion, following activation of BMAL1, contributes to the accumulation of epidermal stem cells [220].

A recent review, in a different branch of work, evaluated the connections between circadian rhythm disorders and carcinogenesis, with particular attention to the ncRNAs involved, which are considered crucial mediators of these mechanisms [221]. The upregulation of miR-192/194 and miR-24/29a/30a has shown a negative correlation with the circadian Period (PER) gene family; miR24/29a/30a also downregulates the expression of PER1 and PER2 and the DNA repair system through the enhancement of the gene expression of CDC2 and c-MYC and the downregulation of the expression of p53. Further to this, miR-219 and miR-132 also target PER1; miR-181d and miR-206 repress CLOCK gene expression; miR-135b acts on the vascular endothelial growth factor (VEGF), and its overexpression reduces the level of BMAL1 and alters the p53 protein pathway, the DNA repair system, the mechanisms of apoptosis and the cell cycle. miR-16 and miR-20a have anti-proliferative activity and can act on circadian genes. miR-139-5p has an important role in the modulation of TIMELESS, in the response to DNA damage and in maintaining the length and integrity of telomeres. Finally, let-7e-5p and miR-125b-5p act on PER1 and CLOCK genes [221].

Notably, although p53 seems to play an important role in cancer and neurodegenerative diseases, to date, the ncRNAs that regulate p53 have not been investigated in detail. A recent study aimed to identify lncRNAs associated with AD using a human neuroblastoma cell line (SH-SY5Y) treated with Aβ, as a model of this disease [222]. The authors demonstrated that differentially expressed genes were predominantly involved in different pathways, including p53, modulating the cell cycle, post-translational protein modification, and regulation. Approximately 100 dysregulated lncRNA transcripts were found in Aβ-treated SH-SY5Y cells, suggesting that these lncRNAs may play an important role in the occurrence and development of the AD pathology through altered signal pathways [222]. In this context, it was previously found that Aβ precursor-like protein (APLP1) was a novel p53 transcriptional target gene that augmented neuroblastoma cell death on genotoxic stress [223]. In particular, the authors observed that depletion of APLP1 expression reduced the stress-induced apoptosis of neural cells, whereas ectopic APLP1 expression increased apoptosis. Based on these data, a mechanism was proposed whereby p53-dependent induction of APLP1 was involved in neuronal death, which may exacerbate some neurodegenerative disorders [223]. Finally, the polo-like kinase 2 (PLK2) is highly expressed in cells with defective mitochondrial respiration and is essential for their survival [224]. Although PLK2 has been widely studied as a cell cycle regulator, it has also been found that expression of PLK2 is responsive to oxidative stress and that the antioxidant activity of PLK2 is essential for preventing p53-dependent necrotic cell death [224]. Thus, the regulation of redox homeostasis by PLK2 can promote the survival of cells with dysfunctional mitochondria, which may have therapeutic implications for both cancer and neurodegenerative diseases. The function of lncRNAs could be mediated by miRNAs or independently through transcriptional or epigenetic regulation [81]: miR-26a appears to be activated by both CLOCK and CREB (cAMP-response element-binding protein-1) [221]. Another lncRNA, telomeric repeat-containing RNA (TERRA), which has an important role in preserving telomeres and in tumor processes, as well as in the stability of the human genome [225], has been associated with BMAL1 [226]. On the other hand, the deletion of BMAL1 deregulates the diurnal rhythmic expression of TERRA in the lungs [227].

A recent study has shown that the overexpression of MINCR (MYC-induced long noncoding RNA) causes significant alterations in cancer-related genes, inducing alterations to the cell cycle and the signaling of growth factors; on the contrary, the downregulation of MINCR influences a small number of genes involved in various neurodegenerative disorders, mainly concerning metabolism and RNA inflammation [82]. At the moment, there are no studies in the literature that have indicated a connection between MINCR and circadian genes, although further research on this would be desirable in the light of the evidence found, as well as research considering its role in the upregulation of RTKN for the activation of the Wnt/β-catenin pathway. This signaling pathway is upregulated in carcinogenesis and downregulated in neurodegenerative disorders [32], and circadian rhythms play a key role in its modulation [228].

The PI3K/Akt/mTOR pathway is involved in some neurodegenerative and tumor processes; moreover, mTOR has a significant role in the modulation of autophagy induction and is inversely related to SIRT1 (the latter is involved in neurodegenerative and tumor processes). Finally, both mTOR and SIRT1 are involved in the regulation of the expression of circadian genes [229]. Activation of the PI3K/Akt/mTOR pathway is associated with potentiation of miRNA-7 and miRNA-221 in Parkinson’s disease (PD), showing promising neuroprotective effects [230]; conversely, the overexpression of PI3K partially inverses the effects of miR-7 on cell growth inhibition and cell cycle arrest in glioma cells, while miR-221 and miR-222 reduce viability and induce apoptosis in gastrointestinal stromal tumors [231]. Therefore, the different means of modulation of miRNA-7 and miRNA-221 can act on the PI3K/Akt/mTOR pathway in some neurodegenerative and tumor processes, while, at the cellular level, the regulation of autophagy pathways mTOR, AMPK and SIRT1 may be vital for the normal regulation of circadian rhythms, [229] which, in turn, is of vital importance for preventing tumor and neurodegenerative pathologies.

### 6.3. Future Therapeutic Perspectives

It is, therefore, evident that the study of the inverse comorbidity of miRNAs in neurodegenerative and tumor processes, associated with correlations with circadian genes, can further narrow the field of molecular pathways to be used as a therapeutic target.

In fact, in recent years, it has emerged that environmental and lifestyle changes affect circadian rhythms, and that it is, therefore, important to devise therapeutic strategies based on biological clocks, such as chronotherapy, in which the time dosage of the drug is optimized for the maximum index. In this way, therapeutic and pharmacological agents target the components of the biological clock. At the moment, promising advances in chronotherapy have been highlighted in different areas: cancer, neurodegenerative diseases (AD), stroke and myocardial infarction, asthma, inflammatory disorders and metabolic syndrome. Chronotherapy works through modulation of different target pathways: REV-ERBs, RORs, PER, CRY and CK1 (kinases that play an important role in the phosphorylation of clock gene proteins) [232].

Among the epigenetic mechanisms that control circadian rhythms, miRNAs are the least studied, despite their promising therapeutic implications; several studies have been conducted on animals (mouse models and *Drophosila melanogaster* models), but very few on humans. A recent study has shown that miRNAs can also control circadian genes in humans, demonstrating daily variations in the expression of miR-16 and miR-181 in human leukocytes (both peaking between 8:00 am and 04:00 pm) [233]. miR-181 has been associated with glioblastoma and lipid metabolism [234], and as previously described, is involved in neurodegenerative processes such as AD [218], and it has an antiproliferative action in carcinogenesis [221], representing a therapeutic option in both cases. A recent review also highlighted a role for mi-RNA 125, implicated in carcinogenetic processes, in the modulation of some circadian genes, with a possible use in chronotherapy [235].

Furthermore, drugs that specifically target lncRNA TERRA molecules could modulate telomerase-mediated elongation of telomeres, thus representing a promising therapeutic strategy against cancer and age-related diseases [236].

## 7. Conclusions

**Figure 5:**
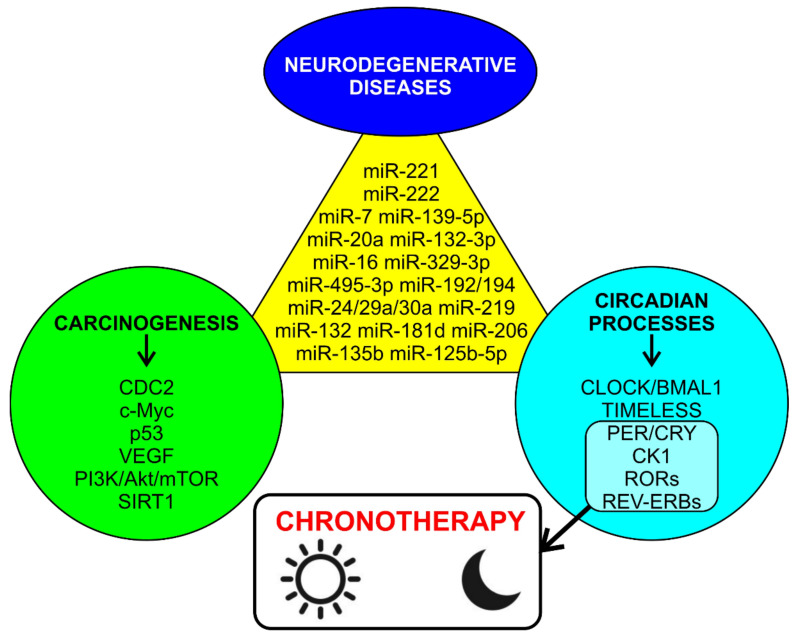
miRNAs involved in inverse comorbidity mechanisms for the modulation of neurodegeneration, carcinogenesis and circadian genes.

miRNA profiling represents a powerful tool to differentiate tumors from normal tissues and classify cancer subtypes [204,205,206]; in contrast, miRNA deregulation represents a common feature in cancer, caused by several mechanisms, such as deletion, amplification, chromosomal rearrangements and epigenetic regulation at various levels [207]. miRNAs are frequently localized to fragile sites and genomic regions involved in tumors [207]; this could be key to better understanding their role in tumor genesis and also in neurodegenerative mechanisms by acting on specific target genes, corroborating the growing data in the literature highlighting these reverse comorbidity mechanisms (Figure 5). MiR-519a-3p and one of its target genes, *PARP1,* provide an example. Although miR-519a-3p and *PARP1* move in opposite directions in terms of expression in the mentioned tumors compared to PDs, this does not mean that this model can be applied to all tumors or other neurodegenerative diseases. In any case, we believe that *PARP1* may play a pro-apoptotic role in neurodegeneration by destroying the affected neurons, which leaves less evidence of *PARP1* in the neurons, as indicated by immunofluorescence, as it is no longer present and detectable. In the context of the tumors mentioned above, we cannot rule out the possibility that overexpression is the “extrema ratio” for *PARP1* to drive tumor cells toward apoptosis.

ncRNAs showing reverse comorbidity between PCa and neurodegenerative diseases are also important. Indeed, ncRNAs could represent a very important tool to better understand the mechanisms underlying PCa and neurodegenerative diseases, as well as potentially becoming prognostic markers and therapeutic targets. Although further studies are needed, we have shown that microRNAs are of primary importance in the circadian regulation of cellular physiology, as well as when considering the reverse comorbidity mechanisms between cancer and neurodegenerative disorders.

On the other hand, in the field of precision medicine, the use of epigenetics (including the use of miRNAs) offers important diagnostic, prognostic and therapeutic perspectives in a growing range of medical areas, for targeted therapies “ad personam” and increased bioavailability of drugs at the required anatomical sites, among other benefits, inducing fewer side effects.

We believe that once the functions of all genes are better understood, we should comprehensively address all regulatory mechanisms acting on these genes. If we consider that only a small part of DNA (about 2%) encodes genes, the remaining 98%, if left in the evolutionary process, will play an important role in the regulation of the genes themselves. Thus, a challenge for future research is how to carefully study all the transcribed RNAs that do not translate proteins. Certainly, ncRNAs are important players in terms of how we can better address the two major areas of human disease approached in this review, i.e., cancer and neurodegenerative disorders.

## Data Availability

Not applicable.
